# First Description of Loreto Virus in Three Culicidae Species from the Atlantic Forest, Bahia, Brazil

**DOI:** 10.3390/v16111674

**Published:** 2024-10-25

**Authors:** Thito Y. Bezerra da Paz, Leonardo H. Almeida Hernández, Fábio Silva da Silva, Ana C. Ribeiro Cruz, Sandro Patroca da Silva, Hellen Thais Fuzii, Janaina M. Vasconcelos Massafra, João L. S. G. Vianez Júnior, Sharon L. Deem, Leonardo de Carvalho Oliveira, Kristel Myriam De Vleeschouwer, Lilian Silva Catenacci

**Affiliations:** 1 Parasite Biology in the Amazon Region Graduate Program, Pará State University, Belém 66087-670, PA, Brazilanacecilia@iec.gov.br (A.C.R.C.); 2 Department of Arbovirology and Hemorrhagic Fevers, Evandro Chagas Institute, Health and Environmental Surveillance Secretariat, Ministry of Health, Ananindeua 67030-000, PA, Brazil; 3 Tropical Medicine Center, Federal University of Pará, Belém 66055-240, PA, Brazil; 4 Center for Technological Innovation, Evandro Chagas Institute, Health and Environmental Surveillance Secretariat, Ministry of Health, Ananindeua 67030-000, PA, Brazil; 5 Saint Louis Zoo Institute for Conservation Medicine, Saint Louis, MO 63110, USAcatenacci@ufpi.edu.br (L.S.C.); 6 Department of Sciences, Faculty of Teacher Training, Rio de Janeiro State University (FFP-UERJ), Rio de Janeiro 24435-005, RJ, Brazil; 7 Centre for Research and Conservation, Royal Zoological Society of Antwerp, 2018 Antwerp, Belgium; 8 Departament of Veterinary Morphophisiology, Applied Technologies for Regional Animals (PPGTAIR), Federal University of Piauí, Teresina 64049-550, PI, Brazil; 9 Animal Health in the Amazon Graduate Program (PPGSAAM), Federal University of Pará, Castanhal 68746-630, PA, Brazil; 10 Center for Intelligence on Emergent and Neglected Tropical Diseases, Federal University of Piauí, Teresina 64049-550, PI, Brazil

**Keywords:** negevirus, nelorpivirus, insect-specific viruses, next-generation sequencing

## Abstract

Loreto virus (LORV) is an insect-specific virus classified into the proposed taxon Negevirus. It was originally described in Iquitos, Peru, in 1977. Here, we describe three novel LORV genomes obtained from the isolates of three pooled samples of *Trichoprosopon digitatum*, *Aedes* (*Ochlerotatus*) *fulvus,* and *Limatus durhamii* collected in Ilhéus—Bahia, 2014. Samples were submitted to RNA sequencing on the Illumina platform to recover the LORV genome. The genomes presented, on average, 81.5% nucleotide identity and 92.6% global amino acid identity with the LORV reference genome (NC_034158). Subsequently, phylogenetic analysis was performed based on a multiple sequence alignment of the concatenated amino acid sequences predicted for the three ORFs of the Negevirus genomes, and the target sequences were clustered within the LORV clade. The taxon Negevirus is in constant expansion of its species content and host range. New data about insect specific negeviruses are important for virus evolution studies, along with those approaching interactions with the hosts and their influence in the transmission of arboviruses. Also, the assessment of these data may allow the development of biologic control strategies for arboviral vectors. This is the original report of the identification of LORV in Brazil, infecting three Culicidae species hosts native to the Atlantic Forest biome.

## 1. Introduction

The Loreto virus (LORV) is classified into the proposed Negevirus taxon, composed of insect-specific viruses (ISVs) presenting a single-stranded positive-sense RNA genome of approximately 9 to 10 kb in length and spherical viral particles of 45 to 55 nm in diameter. Their diversity and host range are in constant expansion [1]. Negeviruses seem to be ubiquitous to several biomes, being isolated from various geographic locations in the Americas, Africa, the Middle East, Europe, Asia, and Australia [2].

The LORV strains 2617/77 and PeAR 2612/77 were originally isolated from *Lutzomyia* spp. and *Culex* spp. pools, respectively, and both were collected in Iquitos, Peru, in 1977. Later, in 1983, the strain 3940-83 was isolated from *Anopheles albimanus*, collected in Lima, Peru [1]. Recently, the negeviruses Brejeira, Negev, Cordoba, Wallerfield, and the tentatively named Feitosa virus were isolated from *Mansonia* spp. mosquitoes in the Brazilian Amazon [3]. To the authors’ knowledge, here we report the first detection of the Loreto virus in Brazil and its association with three new Culicidae host species native to the Atlantic Forest and not previously found infected with the virus. It is also the first report of the virus outside the Amazon biome.

## 2. Materials and Methods

### 2.1. Sample Collection

**Figure 1:**
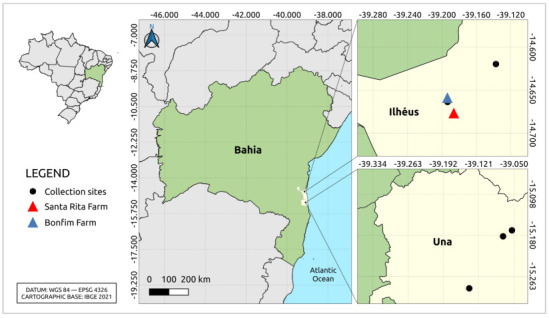
Collection sites (black dots) of the mosquito samples in Atlantic Forest fragments in Ilhéus and Una municipalities—Bahia state, Brazil. Collection sites of the positive samples (Santa Rita and Bonfim farms) are highlighted in red and blue. More details are provided in the Supplementary Materials (Table S1).

Mosquito samples were collected for an entomo-virological survey in Atlantic Forest fragments in Bahia state, Northeastern Brazil, at multiple locations (Table S1), which were part of an ongoing arbovirus surveillance program in Ilhéus and Una municipalities (Figure 1), from 2009 to 2014. The collection sites included natural areas (bushland and forest) and forest mosaic with some human presence. The predominant land uses in the region of study are cacao agroforests, rubber tree plantations, and coconut and cassava plantations [4,5].

The diurnal capture was carried out by employing manual collection using manufactured hand nets at the ground and canopy levels, and nocturnal collection was carried out with non-baited CDC light traps at the ground level. The specimens were identified by morphological features according to the Consoli and Oliveira dichotomous keys [6] and grouped on 304 pools based on date, site of collection, and taxonomic identification.

### 2.2. Virus Isolation in Cell Culture

A suspension was prepared with the addition of 1 mL of D-PBS 1× diluent (Gibco, Waltham, MA, USA), 2% penicillin and streptomycin, 1% fungizone, and 5% FBS to the samples. The mechanical maceration was performed with the addition of a 3 mm tungsten bead in a TissueLyser II system (Qiagen, Hilden, Germany) and grinding at 25 Hz for 1 min. The samples were centrifuged at 10,000× *g* for 10 min at 4 °C, and 100 μL of the supernatants were inoculated in C6/36 cell cultures for viral isolation.

The *Aedes albopictus* clone C6/36 cell line (ATCC: CRL 1660) was maintained at a temperature of 28 °C in the Leibowitz L-15 medium with L-glutamine (Gibco, Waltham, MA, USA) supplemented with 5% fetal bovine serum (FBS) (Gibco, Waltham, MA, USA), to which 2.95% tryptose phosphate (Himedia, Mumbai, India), antibiotics (penicillin 10,000 U/L and streptomycin 10,000 g/L) (Gibco, Waltham, MA, USA), and non-essential amino acids (10 mL/L) (Baktron Microbiology, Rio de Janeiro, RJ, Brazil) were added. The inoculated samples were then observed daily for seven days under an inverted microscope to visualize any cytopathic effect and other abnormalities in the cell monolayer.

### 2.3. RNA Extraction and Sequencing

The RNA extraction was performed using 140 µL of the supernatants of the cell cultures after cytopathic effects were observed on the 6th day post-inoculation; it was performed using the kit Qiamp Viral RNA Mini Kit (Qiagen, Hilden, Germany), followed by the complementary DNA (cDNA) synthesis using SuperScript VILO™ Master Mix (Thermo Fisher Scientific, Waltham, MA, USA) for the first strand synthesis and the NEBNext^®^ mRNA Second Strand Synthesis Module (New England BioLabs, Ipswich, MA, USA) for the second strand synthesis.

The cDNA library for shotgun sequencing was prepared using the Nextera XT DNA Library Preparation Kit (Illumina, San Diego, CA, USA). Quantification and fragmentation level assessment were performed using a Qubit 2.0 Fluorometer (Thermo Fisher Scientific) and a 2100 Bioanalyzer Instrument (Agilent Technologies, Santa Clara, CA, USA) prior to sequencing on the MiSeq platform (Illumina) and applying the MiSeq Reagent kit (version 3) (150 cycles) with a paired-end (2 × 75 bp) protocol. All procedures were performed according to the manufacturer’s instructions.

### 2.4. Sequencing Analysis

All the following analyses were performed using the software’s default parameters. The output files were filtered for quality control to remove adapters and redundant reads, and ribosomal RNA sequences were discarded for analysis with the software TrimGalore v.0.4.5 [7], CD-HIT v.4.8.1 [8], and SortMeRNA v.2.1 [9], respectively. The reads were de novo assembled with IDBA-UD and aligned with DIAMOND v.1.2.9 [10] against the non-redundant proteins database—NR (NCBI). The resulting file was visualized using MEGAN6 [11]. The contigs corresponding to LORV were extracted and inspected at Geneious Prime [12], where they were aligned against the reference genome (NC_034158), assembled in a consensus sequence, and manually annotated. The three sequences were submitted to the NCBI nucleotide database under the accession numbers OR829950 to OR829952.

A sliding window similarity analysis was conducted using the Simplot++ v.1.3 with the input of a multiple sequence alignment containing the novel LORV genomes and the reference genome and applying the Jukes–Cantor model to estimate the similarity between them in a window size of 200 nt with intervals of 20 nt.

Additionally, a dataset comprising the concatenated amino acid sequences of the three open reading frames (ORFs) of representants of the Nelorpivirus and Sandewavirus clades was submitted to a multiple sequence alignment using ClustalW. IQ-TREE2 v.2.3.1 [13] software was used for the prediction of the best-fit model to obtain the phylogenetic signal for the aligned dataset and for the construction of the maximum likelihood phylogenetic tree by applying the bootstrap test with 1000 replicates. 

## 3. Results

From the inoculated samples, 63 presented a cytopathic effect (CPE). Here, we focus on the data obtained from the isolates of three pooled samples of *Trichoprosopon digitatum* (9 specimens), *Aedes (Ochlerotatus) fulvus* (2), and *Limatus durhamii* (30) collected in Ilhéus—Bahia in 2014. The described samples were arbitrarily chosen to initiate the processing. Altogether, the sequencing run generated 12,024,573 reads. The inspection of the reads and contigs revealed sequences for which the highest identities were with LORV.

The contigs were mapped against the reference genome of LORV (NC_034158), generating three nearly complete genomes preserving the overall genome architecture but lacking the terminal part of the ORF 3. The genome coverage metrics for the sequences are provided in Table S2.

However, some features were slightly distinct in these new genomes, as the length of the intergenic region between the ORFs 2 and 3 had a 24 nt insertion that seemed to be the result of the duplication of the first eight codons for the hypothetical protein 3 (ORF 3 product), as shown in Figure S1. There was also a single-nucleotide deletion in position 7163 of the reference genome.

**Figure 2:**
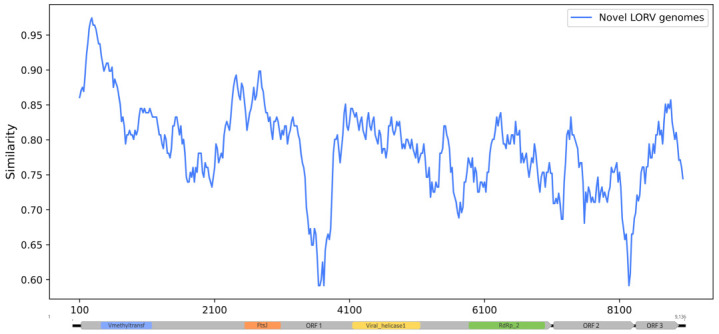
Sliding window Simplot graph showing a genome-wide comparison between the three novel LORV genomes and the reference genome (NC_034158). The corresponding protein domains in ORF 1 are highlighted (Vmethyltransf: viral methyltransferase; FtsJ: FtsJ-like methyltransferase; Viral_helicase1: Viral (Superfamily 1) RNA helicase; RdRp_2: RNA-dependent RNA polymerase.

The genomes were almost identical, with only two synonymous nucleotide substitutions in the OR829952 genome. The sequences exhibited 81.47–81.54% nucleotide identity and 92.6% global amino acid identity with the reference genome. Table 1 shows the identities between each ORF and the reference sequence of LORV. The sliding window Simplot graph showed a minimum similarity index of approximately 0.6 in portions of ORF 1 and ORF 2, as shown in Figure 2.

**Figure 3:**
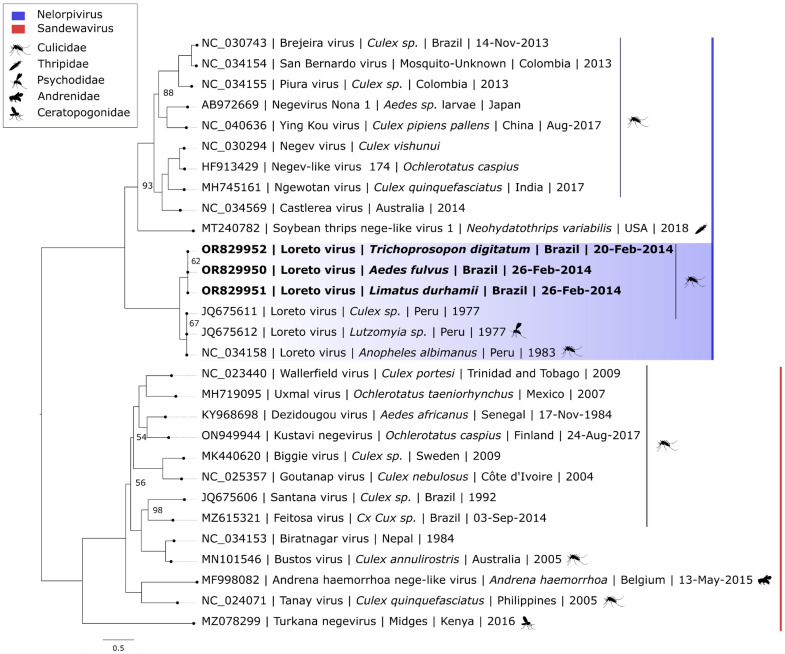
Phylogenetic inference with representants of the Nelorpivirus and Sandewavirus taxa showing the positioning of the novel LORV genomes in a distinct clade from the original sequences. Only bootstrap values under 100 are shown.

The best-fit model determined for the phylogenetic inference was GTR + F + I + R4, and the phylogenetic signal was 86.6% (Figure S2). The reconstruction positioned the novel strains in a monophyletic clade with the original sequences of LORV within the Nelorpivirus taxon, although clustering together in a distinct subclade (Figure 3).

## 4. Discussion

The present study describes three new isolates of LORV with relatively high nucleotide divergence (≈81.5%). As a comparison, the Manglie virus, isolated from *Culex tritaeniorhynchus* in China, showed 79% nucleotide identity with the Ngewotan virus and was proposed to be a new species in the taxon [14]. However, the Negevirus taxon is not yet recognized by the International Committee on Taxonomy of Viruses (ICTV), and there is an absence of identity value thresholds for the species demarcation criteria.

The formation of a new LORV subclade in the phylogenetic analysis reiterates the considerable genetic variability within this viral group, which could be both a result of the geographical origin of each strain and/or the 37-year time span since the collection of the original LORV genome. The elucidation of the mechanisms driving the genetic diversity among LORV strains requires the acquisition of new viral genomes to compose a comprehensive dataset, which would allow for more precise inferences.

Since the proposition of the taxon, the number of negeviruses and related sequences detected in metagenomic studies has significantly increased. These viruses are abundant entities in the virome of a plethora of arthropod species, and many of them have already been detected in the Brazilian territory, mainly in the Amazon region but also in the Pantanal biome [15].

The discovery of negeviruses was initially biased towards arthropod vectors of arboviruses; however, this issue is being overcome by the sampling of distinct taxa of invertebrate organisms [16]. Also, Nege-like viruses have been detected in a multitude of invertebrate and plant hosts, and their characterization may allow a better resolution of the phylogenetic relationship between the classic negeviruses (Sandewavirus and Nelorpivirus) and the related families of plant viruses *Kitaviridae* and *Virgaviridae* [17,18].

A structural analysis of the Tanay virus revealed this putative species is uniquely shaped, forming an elliptical particle with a projection formed by the glycoproteins. According to the study, some of its structural characteristics may reinforce the relationship between negeviruses and plant viruses, even though no evidence of insect negevirus infection in plant hosts has been found to date [19].

Environmental and climatic conditions seem to influence the geographical distribution of negeviruses, which have only been detected or isolated between latitudes 42° N and 42° S. Despite the many arthropod species (mainly mosquitoes, sand flies, biting midges, and others) associated with this viral group, their maintenance cycles in nature and mechanisms of transmission remain unknown. LORV is one of the two negeviruses described that infect sand flies, along with the Piura virus; however, both viruses appear to have a relatively broad host range, also infecting Culicinae and Anophelinae mosquitoes [20].

Knowledge about the maintenance of negeviruses in Culicidae in nature is limited. Infection through food sources is unlikely to happen due to the high viral titers demanded for oral infection, and vertical transmission would not be the primary source of infection, considering their host range [1]. LORV has now been reported in six distinct host species, which reinforces the need for further investigations on negevirus transmission.

To date, studies about their circulation in other Brazilian biomes are scarce, and this is, to our knowledge, the first description of the LORV in Brazil, in the Atlantic forest, and in these three Culicidae species (*Trichoprosopon digitatum*, *Aedes (Ochlerotatus) fulvus*, and *Limatus durhamii*), all of which have already been associated with arbovirus transmission or natural infection, reinforcing the sampling bias on negevirus discovery and detection towards arboviral vectors.

The genus *Limatus* (Diptera: Culicidae) currently comprises nine species of sylvatic mosquitoes and has a wide Neotropical distribution from central to eastern South America and the Caribbean islands [21]. These mosquitoes breed in a variety of natural habitats, as phytotelmata, and in artificial water containers. Immature mosquitoes are saprophagous but may develop predatory behavior on other larvae in scarce environments [22]. The *L. durhamii* species is suspected of participating in the transmission of arboviruses from the *Orthobunyavirus* genus. This is the first association of the species with a negevirus.

*Ae. fulvus* breeds on transitory flooded areas and perennial wetlands with vegetation. Their blood meal sources include humans; however, their behavior is mostly zoophilic. Predominantly, these mosquitoes are found in better-preserved forests [6,23] but also in peridomestic environments [24]. Their activity is constant during the day and night hours but more intense at crepuscular hours and at the ground and canopy levels. This species was found naturally infected with the yellow fever virus (*Orthoflavivirus flavi*), Puerto Almendras virus (*Almendravirus almendras*), Melao virus (*Orthobunyavirus melajoense*), Eastern Equine Encephalitis virus (*Alphavirus eastern*), and Venezuelan Equine Encephalitis virus (*Alphavirus venezuelan*) [6,25].

*Tr. digitatum* is one of the most widely distributed species from Mexico to the most southern limit in São Paulo state, Brazil. These sylvatic mosquitoes can be commonly found in altered environments, such as plantations and peridomiciles, and have zoophilic and anthropophilic feeding behavior that intensifies at crepuscular hours. Their breeding sites are mainly on fruit peels and bamboo holes. The Pixuna (*Pixuna virus*), Bussuquara (*Orthoflavivirus aroaense*), Wyeomyia (*Orthobunyavirus wyeomyiae*), and Ilheus (*Orthoflavivirus ilheusense*) viruses have already been associated with this species [6,26].

Considering the expressive host range of negeviruses in nature, surveys for negeviruses, such as LORV, should also be carried out by specific methods such as RT-PCR [27], which is more affordable, and in a variety of other hosts to access the actual distribution of these viruses in nature. Along with viral discovery studies, these efforts would also help to elucidate the mechanisms of their maintenance cycles and, consequently, enlighten the evolutionary basis for their ecological traits.

The development of biotechnological applications for ISVs is promising. Studies regarding their use as a biological control for the replication of arboviruses in mosquito vectors are being developed, although the interaction between ISVs and closely related arboviruses (i.e., belonging to the same family) has been more intensely explored [28]. However, many studies aiming to elucidate the dynamics of the co-infection between negeviruses and arboviruses, mainly from the *Flaviviridae*, *Peribunyaviridae*, and *Togaviridae* families, demonstrate a negative regulation of the replication of several arboviruses in vitro [29,30].

## 5. Conclusions

Efforts to address the lack of knowledge about ISV diversity in the Americas, including their geographical distribution, ecology, and genetic variation, are essential to unravel their biotechnological potential. Viral discovery research has provided a lot of novel sequences of negeviruses to the repertoire of ISVs that can be used for a variety of useful applications for public health involving arthropod-borne diseases. As a follow-up to our description of this novel strain of LORV, we recommend that future research focuses on the characterization of this isolate and on elucidating the effects of its long-term evolution, both of which may be valuable for better understanding the dynamics of negevirus infection in arthopods.

## Figures and Tables

**Table 1:** Nucleotide (nt) and amino acid (aa) identities (%) across the three ORFs of the novel LORV genomes and the reference genome (NC_034158).

		Identities (%)					
		ORF1		ORF2		ORF3 *	
Sample	Host	nt	aa	nt	Aa	nt	aa
OR829950	*Ae. fulvus*	82.549	93.753	77.363	87.531	82.439	92.683
OR829951	*L. durrhamii*	82.563	93.753	77.363	87.531	82.771	91.979
OR829952	*Tr. digitatum*	82.563	93.753	77.363	87.531	82.343	92.574

* The difference between the identities may reflect the incompleteness of the ORFs, given the overall similarity between the genomes.

## Data Availability

The sequences regarding this publication are available at GenBank under the accession numbers OR829950 to OR829952.

## Supplementary Materials

The following supporting information can be downloaded at: https://www.mdpi.com/article/10.3390/v16111674/s1, Table S1: Coordinates of the collection sites; Table S2: Genome coverage metrics; Figure S1: Multiple sequencing alignment highlighting the duplication of the eight initial codons of ORF 3; Figure S2: Graphical representation of the phylogenetic signal.
